# Factors in Color Fundus Photographs That Can Be Used by Humans to Determine Sex of Individuals

**DOI:** 10.1167/tvst.9.7.8

**Published:** 2020-06-05

**Authors:** Simon Dieck, Miguel Ibarra, Ismail Moghul, Ming Wai Yeung, Jean Tori Pantel, Sarah Thiele, Maximilian Pfau, Monika Fleckenstein, Nikolas Pontikos, Peter M. Krawitz

**Affiliations:** 1 Institute for Genomic Statistics and Bioinformatics, University Bonn, Bonn, Germany; 2 UCL Cancer Institute, University College London, London, UK; 3 Faculty of Medical Sciences, University Groningen, Groningen, Netherlands; 4 Department of Ophthalmology, University Bonn, Bonn, Germany; 5 Department of Ophthalmology and Visual Science, University of Utah, Utah, USA; 6 UCL Institute of Ophthalmology, University College London, London, UK; 7 Institute of Human Genetics and Medical Genetics, Charité - Universitätsmedizin, Berlin, Germany; 8 Department of Biomedical Data Science, Stanford University, Stanford, California, USA

We were intrigued by the findings of Yamashita et al*.* in their recent paper “Factors in Color Fundus Photographs That Can Be Used by Humans to Determine Sex of Individuals.”^1^ As mentioned by the authors, the publication by Poplin et al. in 2018 captivated the interest of many ophthalmologists when the authors proposed a deep neural network for sex differentiation on color fundus photography (CFP) with an area under the curve (AUC) of 97%.^2^ However, to date, the results have yet to be replicated, the neural network model was not shared for independent validation nor explained in detail, and the published saliency maps did not highlight any specific features except for the fovea and the optic disc. This has led to speculation as to what features the black box artificial intelligence (AI) of Poplin et al. might have actually used to achieve such high performance.

Of the hypotheses suggested by our colleague ophthalmologists so far, most have been anatomic differences between men and women, including distance of the optic disc from the fovea, thickness of the optic nerve head, thickness of the choroid, and geometric properties of the vasculature, but also genetic, X-linked mosaicism, and even whimsical as whether traces of mascara or eyelashes were being picked up by the AI.

In the work by Yamashita et al., an AUC of 77.9% was achieved for sex differentiation on CFP based on human identified image features when combined in a Ridge binomial logistic regression analysis. The features identified by the authors were optic disc ovality ratio, papillomacular angle, retinal artery trajectory, and retinal vessel angles, the mean red, green, and blue colors of the peripapillary area expressed by a tessellation index.^1^

In order to evaluate AI-based approaches for sex determination on CFP, we have performed meticulous analyses, including the reproducibility of the results from Poplin et al., the evaluation of a deep learning (DL) approach for assessing the informative relevance of previously known and human interpretable features compared to unknown/unstructured image information, and, finally, assessment of human performance against that of AI.

We were able to reproduce the findings of the Poplin et al. 2018^2^ model with respect to sex classification, achieving an accuracy of 82.9%. We implemented a two-class deep convolutional neural network (CNN) and are accordingly using accuracy as an evaluation score. Our model was trained on 70% of the UK Biobank fundus images and tested on 10% with the remaining 20% being used for validation during training.^3^

The training data was augmented and pre-processed using a normalization procedure suggested by Benjamin Graham for classifying diabetic retinopathy as well as random resizes, crops, and flips as commonly used for convolutional neural networks (Figs. 1A, 1B).^4^^,^^5^ The normalization we performed was a weighted subtraction of a Gaussian blur over the color channels. An occlusion screen, explained in more detail later on, can be used to visualize which regions of the image looked more male or female for the AI (Fig. 1C). We have made our model available online at https://github.com/migueLib/fundus2sex.

**Figure 1.**
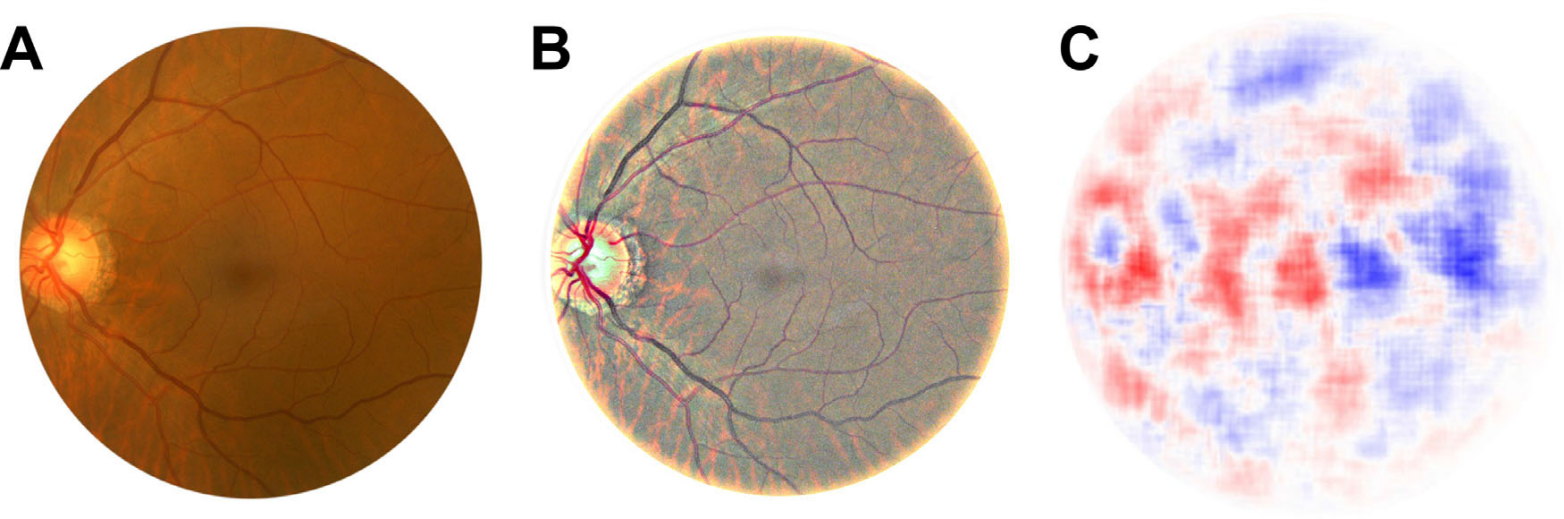
Color fundus photography (CFP) of a female individual before (**A**) and after (**B**) normalization. Occlusion maps indicate which parts of the CFP have a stronger signal for males (blue) and females (red).

The differences in performance between our model and that of Poplin et al*.* might be due to a different experimental setup and a different composition of the training and test data. Poplin et al*.* identified sex inference as a side result in a multiclass problem and their evaluation is based on AUC. In addition to the UK Biobank (UKBB) data, Poplin et al*.* also had access to retinal photographs from EyePACS, a US-based teleretinal services provider and their model was, therefore, trained on more data than ours.

Like Yamashita et al*.*, we tested different hypotheses for sex inference. Besides the features that were already discussed in Yamashita's work, we also analyzed whether the random inactivation of the X-chromosome that occurs in all females with a normal karyotype (46,XX), results in a sex-specific distribution of the photoreceptors.^6^^,^^7^ This is a phenomenon wherein different expressions of genes on the X-chromosome lead to distinct patterns in females due to only one X chromosome being active in each cell. In contrast to the approach of Yamashita et al*.* with a regularized binomial logistic regression model, we used our CNN as a readout. For random X inactivation, for instance, we compiled a test set of 36 CFPs from individuals with Turner syndrome (45,X). However, we had to reject our hypothesis as 33 of 36 cases were classified as female and, therefore, no significant differences in female classification between 45,X and 46,XX could be identified.

Our approach also allowed us to screen for potentially novel anatomic features by subsequently occluding parts of the test image. Gray squares of different sizes were subsequently centered on each pixel of the CFP and evaluated by the network. With this, changes in classification score can be recorded pixel wise and combined into one picture. If a feature important for classification is occluded, a significant change in classification score is expected. This hypothesis-free, unsupervised approach results in an occlusion map that is comparable to a saliency map known from network backpropagation analysis. However, in contrast to backpropagation, occlusion tests do not depend on the network architecture and quality does not deteriorate with multiple layers.

In occlusion maps for 120 CFPs, we had a strong signal in 80% of the cases around the optic disc and in about 50% also the center of the macula was highlighted (see Fig. 1C). As we could not identify any significant structural differences within the center of the maculae between males and females, we hypothesized that either size, ovality, or the distance to the optic disc could be the significant features. We manually labeled each of the 15,000 pictures with two bounding boxes, one for fovea and one for optic disc, allowing us to test if these features could separate the classes. Linear Discriminant Analysis (LDA) was performed on the bounding box coordinates, the area estimation of the boxes and the L1 norm distance between the center points of the bounding boxes per image. However, LDA resulted in largely overlapping distributions (Supplementary Fig. S1). The labeled CFPs with the coordinates of the bounding boxes can also be found in https://github.com/migueLib/fundus2sex.

The optic disc in contrast shows much more structural variation due to the vessels branching out. By visual inspection of hundreds of CFPs that were correctly classified by the AI and achieved a high classification score, our attention was drawn to the infratemporal artery and vein located in this area. We found a significant correlation between sex and angle between infratemporal and infranasal arteries and veins. Noting that this angle is strongly correlated to the other angles between inferior and superior nasal and temporal veins and arteries, we identified the angle easiest to assess for humans. As such, we used the angle between inferior and superior nasal veins for further human evaluation.

Although we finally came to a similar conclusion as Yamashita et al*.,* that is the angles between vessels as a key anatomic feature for sex inference, it is noteworthy to mention the differences in how this was achieved. Yamashita et al*.* knew a priori about the results of Pope et al*.* that described the female bulbi as more rugby-shaped, which causes the trajectories of superior and inferior arteries and veins to be closer to each other.^8^ In contrast, our attention was drawn to the structure of the vessels by means of occlusion sensitivity analysis (Fig. 2), suggesting that an AI can teach us where to look specifically without domain expertise.

**Figure 2.**
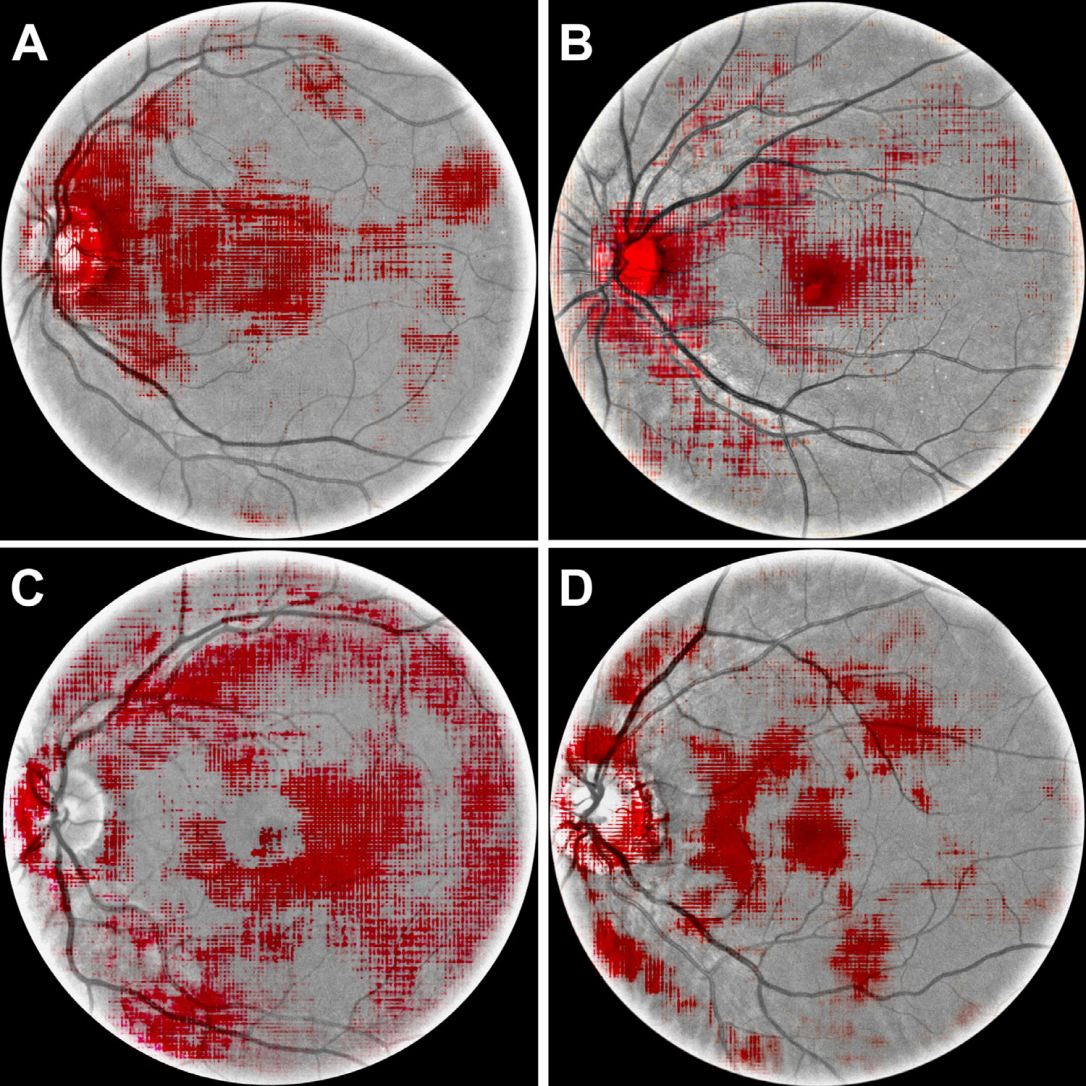
Occlusion maps for two male (**A,** **B**) and two female (**C,** **D**) CFPs. Occlusion maps were thresholded and their contrast enhanced for visualization purposes. Occluding the area around the optic disk affects the classification accuracy most considerably.

However, we did not identify differences in the color between male and female retinas, as described by the tessellation index of Yamashita et al. We believe that this is due to our normalization process that enhanced the contrast of images. This improved the assessment of the angles between retinal veins and arteries as a sex-related feature, for humans as well as the CNN, while reducing the differences in the color distribution and, hence, the tessellation index (Fig. 3).

**Figure 3.**
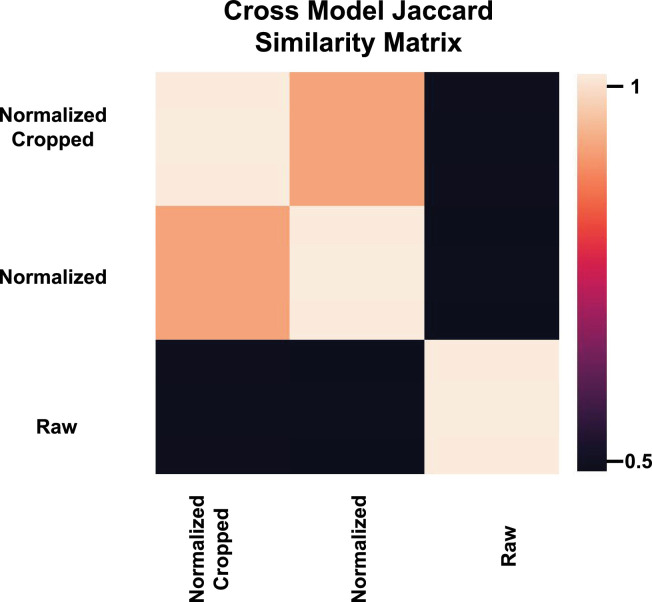
Similarity matrix for the network predictions for raw, normalized, and cropped normalized CFPs using Jaccard index. Normalizing a CFP averages out the differences in color distributions and affects the tessellation index. The low overlap of raw and normalized CFPs indicates that the tessellation index contains meaningful information for sex inference.

In order to assess the performance of humans versus that of AI on this task, we developed an online quiz (www.eye2sex.com). The quiz provided to the user a tutorial on how to discriminate males from females using features visible to the naked eye, such as angle of nasal retinal vessels stemming from the optic disc. The quiz then gave the user 50 images on which to train, where immediate feedback was given, following which 50 test images were given where no immediate feedback was provided but instead a final score was assigned on completion. The final scores were then displayed at the end on a leaderboard. The quiz was completed by 210 participants, which included 30 ophthalmologists and 190 non-ophthalmologists. We found no statistically significant difference in the score of ophthalmologists compared to non-ophthalmologists. We used a Wilcoxon test comparing test result accuracies to a random binomial distribution and found that quiz participants achieved significantly better than random (*P* = 0.0037). However, the mean score on the test was an accuracy of 54%, the ensemble accuracy was 58%, with top performers achieving around 70%. Although we saw significant improvement compared to random, especially for individuals who took the test multiple times, this is still far from the 82.9% achieved by our CNN. Images people consistently got right or wrong could allow us to further refine our rules and instructions to improve on these results (Fig. 4). Interestingly, we did not find any significant correlation in the confidence with which images were classified correctly by human participants compared to the AI.

**Figure 4.**
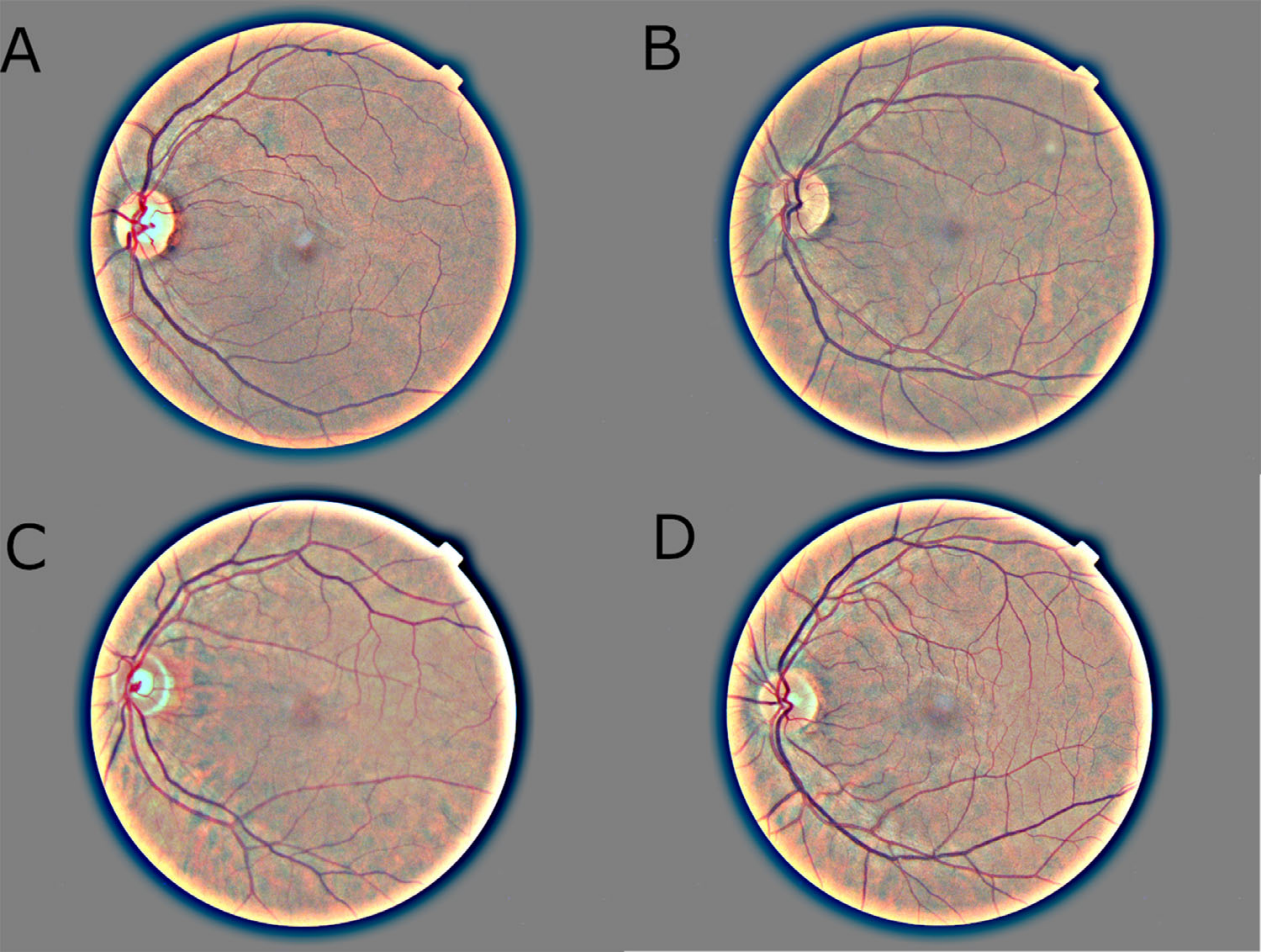
CFPs that were classified by at least seven people and were either always classified correctly (**A**) female and (**B**), male or incorrectly (**C**) female and (**D**) male. **A** and **B**, respectively, show pictures with an easily apparent small or wide angle between nasal veins even with additional arteries present in **B**. **C** shows a pattern of a near horizontal vena superior and a steep vena inferior, which was observed multiple times, possibly forming a secondary pattern for female CFPs. **D** was difficult to classify as it is uncertain whether the central nasal vein is the superior or inferior vein.

While AI uses some known or novel features, as described, the superior performance of AI in sex inference over human participants suggests that there are additional features than those highlighted by Yamashita et al*.* and us, which remain unknown or even unconceivable to us.

## Supplementary Material

Supplement 1
