# Supplementary material for: Factors in Color Fundus Photographs That Can Be Used by Humans to Determine Sex of Individuals
Source: Transl Vis Sci Technol. 2020 Jun 5;9(7):8. doi: 10.1167/tvst.9.7.8 (PMC7414790; doi:10.1167/tvst.9.7.8)
Supplement: Supplement 1 [file tvst-9-7-8_s001.pdf]

Supplemental

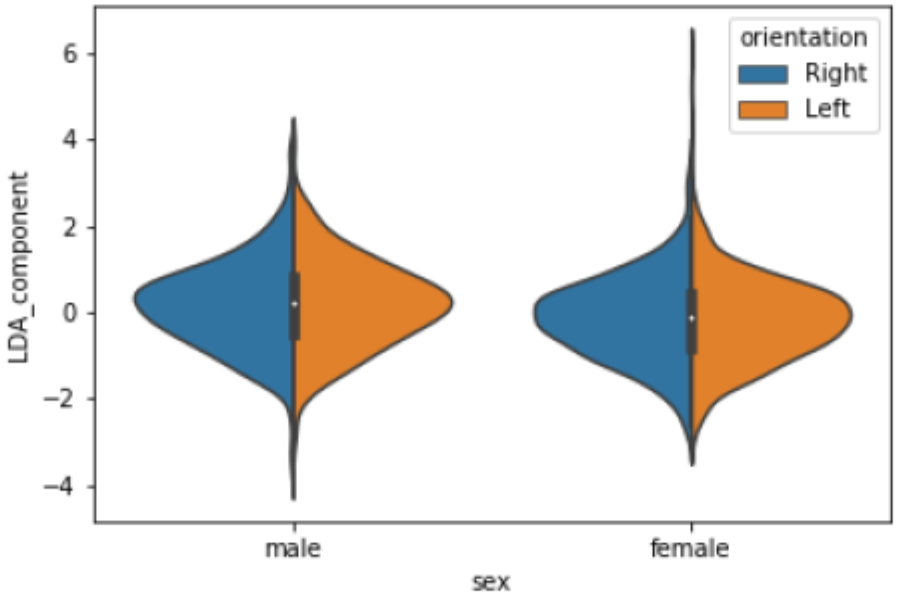

Sup Figure S1. Distributions of male and female samples after a linear discriminant analysis. There is significant overlap between the distributions therefore position, size and distance of optic disk and fovea don't contain enough information to separate the classes. Distributions are displayed separately for left and right eyes.
